# Biomonitoring Heavy Metal Pollution Using an Aquatic Apex Predator, the American Alligator, and Its Parasites

**DOI:** 10.1371/journal.pone.0142522

**Published:** 2015-11-10

**Authors:** Marisa Tellez, Mark Merchant

**Affiliations:** 1 Marine Science Institute, University of California Santa Barbara, Santa Barbara, California, United States of America; 2 Department of Chemistry, McNeese State University, Lake Charles, Louisiana, United States of America; NIEHS/NIH, UNITED STATES

## Abstract

Monitoring the bioaccumulation of chemical elements within various organismal tissues has become a useful tool to survey current or chronic levels of heavy metal exposure within an environment. In this study, we compared the bioaccumulations of As, Cd, Cu, Fe, Pb, Se, and Zn between the American alligator, *Alligator mississippiensis*, and its parasites in order to establish their use as bioindicators of heavy metal pollution. Concomitant with these results, we were interested to determine if parasites were more sensitive bioindicators of heavy metals relative to alligators. We found parasites collectively accumulated higher levels of As, Cu, Se, and Zn in comparison to their alligator hosts, whereas Fe, Cd, and Pb concentrations were higher in alligators. Interestingly, Fe levels were significantly greater in intestinal trematodes than their alligator hosts when analyzed independently from other parasitic taxa. Further analyses showed alligator intestinal trematodes concentrated As, Cu, Fe, Se, and Zn at significantly higher levels than intestinal nematodes and parasites from other organs. However, pentastomids also employed the role as a good biomagnifier of As. Interestingly, parasitic abundance decreased as levels of As increased. Stomach and intestinal nematodes were the poorest bioaccumulators of metals, yet stomach nematodes showed their ability to concentrate Pb at orders of magnitude higher in comparison to other parasites. Conclusively, we suggest that parasites, particularly intestinal trematodes, are superior biomagnifiers of As, Cu, Se, and Zn, whereas alligators are likely good biological indicators of Fe, Cd, and Pb levels within the environment.

## Introduction

With the rise of natural disasters, and industrial and agricultural run-off, the chemical composition of aquatic microhabitats is in a state of transformation that can be a threat to the health and stability of the aqueous biotope [1–6]. Elevated levels of various transition and alkaline earth metals, for example, can hinder the production, maturation, and function of monocytes, which would facilitate the increase susceptibility of an individual or population to pathogens that normally could have been eliminated by the immune system [3, 7–10]. There are various analytical methods to detect fluctuations of heavy metals within the aquatic environment [5]. However, biomonitoring has become a favorable and widely used technique based on the sensitivity of an organisms to subtle current or chronic exposure of heavy metals [5,11,12]. The deleterious effects of altered biochemical and/or physiological states are reflected faster and at lower levels in organisms, which bestows biomonitoring or the use of bioindicators with attractive advantages relative to traditional methods (such as water and soil analyses) [2,5,11–13].

The use of bioindicators can be particularly valuable when comparing heavy metal distribution among populations, geographic locations, or along a temporal scale (between seasons, years, etc.). However, not all organisms are ideal bioindicators for the monitoring of pollutant discharge over an extensive time period. Ideal organisms or species feature a suite of characteristics, such as 1) long life spans, 2) abundant at multiple geographic locations within a well-defined territorial range, 3) occupation of higher trophic levels, and 4) accumulate of high pollutant concentrations without mortality [5,11]. Because of their sensitivity to abiotic fluctuations, and their dependency of multiple hosts in the food web [14], many metazoan parasites of long-lived vertebrates satisfy the criteria of pollutant bioindicators [11,12,15–25]. Likewise, their position as a top trophic consumer [26] can infer cryptic details about the chemical state of the environment as a consequence of food web biomagnifications [11,27].

Results from research investigating the use of parasites as bioindicators have compared the chemical composition between hosts and parasites of secondary or lower trophic level consumers, such as zebra mussels, fish, pigs and cows [11,12,21,22]. At present, knowledge of heavy metal correlation between parasites and reptiles, as well as apex predators, is insufficient. In theory, parasites of a generalist apex predator should demonstrate superior biomagnification capabilities of environmental heavy metal accumulation than secondary or tertiary consumers and their parasites, given the bioaccumulation of pollutants from various trophic links up the food web. Moreover, the importance of bioaccumulation levels could perhaps be further amplified when the predatory host species, such as the American alligator (*Alligator mississippiensis*), is also an ideal sentinel species of pollution. The capacity to readily accumulate heavy metals from prey or their environment [1,28–33], along with their widespread geographic distribution, territorial behavior, longevity, and well-studied physiology and biology, constitute alligators as favorable bioindicators. Thus, the heavy metal analysis of a predator-parasite system, such as alligators and their parasites, could provide a unique perspective concerning heavy metal pollution within the environment.

This is the first study to compare the bioaccumulation of heavy metals in an aquatic, long-lived, apex predator host, the American alligator, and its parasites to determine their role and future use as bioindicators of aquatic pollution. Heavy metal concentrations of four parasitic groups (stomach nematodes, intestinal trematodes, intestinal nematodes, and pentastomids) from three different biological host sites are compared, and contrasted to metal levels found in alligators. We also compared heavy metal levels among geographic locations of alligators and their parasites to determine if host bioaccumulation of metals coincided with levels detected among parasites. Finally, we analyzed the correlation of heavy metal concentrations to parasite abundance in order to examine the possible effects of heavy metals on parasitism. Although we expected alligators to bioaccumulate heavy metals at high levels, we predicted parasites would accumulate pollutants at higher concentrations than their host as observed in other host-parasite systems. As a consequence, alligator parasites could prove to be essential bioindicators of the chemical state of the aquatic ecosystem, which could help foster the appropriate management to prevent further environmental contamination.

## Material and Methods

### Sample Collection and Heavy Metal Analysis Preparation

**Fig 1:**
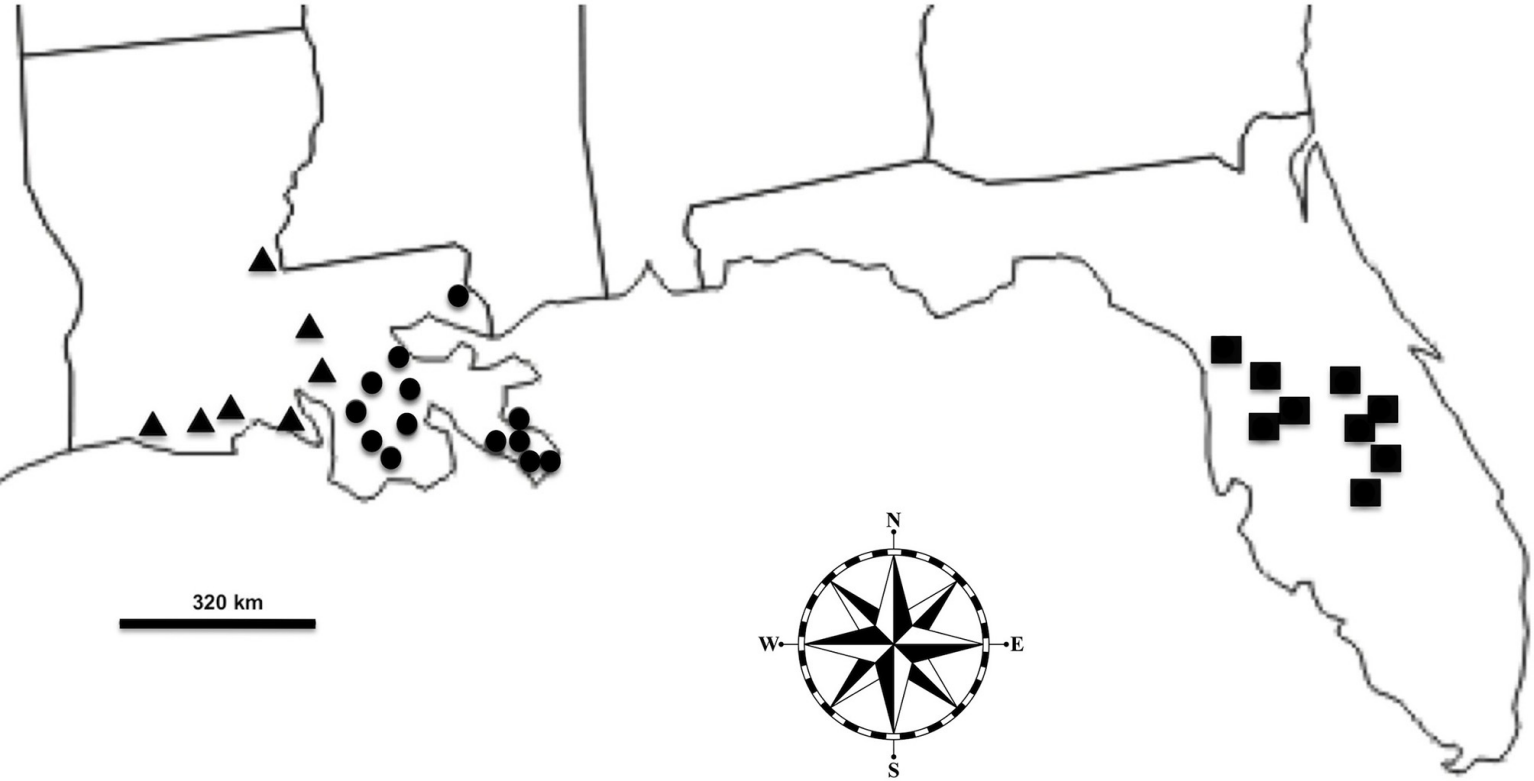
Map of collection sites in Louisiana West Zone (triangles), Louisiana East Zone (circles), and Florida (rectangles).

Alligators were collected with the assistance and permission of the Louisiana Department of Wildlife and Fisheries (LDWF), Florida Fish and Wildlife (FFW), licensed nuisance and annual hunters, and processing sheds for four annual alligator harvests (2009–2012). The alligator harvests of both Florida and Louisiana are internationally recognized programs for the sustainable management of alligator populations. Protected, yet not endangered, the regulated wild alligator harvests provide long-term benefits to the survival of alligators, in addition to providing significant economic and culture benefits to the citizens of the state. In Louisiana, the LDWF Alligator Management Program separates the state into two zones (West Zone vs. East Zone) during the one-month harvest season, which begins in late August. The boundaries of the East Zone (SELA) and West Zone (SWLA) are designated by the LDWF Alligator Management Program, geographically separated by Interstate 55 and the Atchafalaya River. According to the USGS National Land Cover database, LAE sites are characterized by open water, woody wetlands, and big developed intensity, whereas collection sites from LAW are described as cultivated crop and herbaceous wetlands. No boundaries or zones are set in Florida (FL) during the harvest, which begins early August, and ends November 1. Collection sites of Florida alligators are characterized as medium to large development and herbaceous wetlands according to the USGS National Land Cover database. All alligators in our study were collected via legal trapping methods such as legal firearm, hook and line, or bow and arrow, then transported to licensed processing sheds where alligator dissections were performed by our research team in Louisiana and Florida. Geographic origin (Fig 1 and S1 Table), size and sex were recorded of all specimens as this is typical protocol for data recording of crocodilians.

Lungs and stomachs of alligators were removed from the alligator corpse, and immediately examined for parasites during the 2009–2012 alligator harvests. Intestinal tracts were removed from mesentery and either dissected upon removal or put into labeled jars, fixed in 10% formalin, and transferred to the University of California, Los Angeles (UCLA) for dissection. In summary, parasites were recovered from host lungs (SWLA, n = 13; SELA, n = 6; FL, n = 16), host stomach (SWLA, n = 8; SELA, n = 15; FL, n = 26), and host intestines (SWLA, n = 12; SELA, n = 11; FL, n = 4), and stored in labeled vials that were identified to the biological and site of infection, alligator host, and year. Once parasites from lungs, stomachs, and intestines were identified to species (Table 1), they were either stored in an -80°C cooler or in vials of 95% ethanol.

**Table 1:** Summary of parasite species collected from each alligator host/year in each geographic zone.

Zone	Year	Host*	Parasite Species
SELA	2009	13	Ascarid, *Brevimulticaecum tenuicolle*, *Dujardinascaris waltoni*, *Ortleppascaris antipini*
	2010	A	*Dujardinascaris waltoni*
	2010	B	*Dujardinascaris waltoni*, *Ortleppascaris antipini*
	2010	C	*Ortleppascaris antipini*
	2010	H	*Dujardinascaris waltoni*
	2010	I	*Dujardinascaris waltoni*
	2010	J	*Dujardinascaris waltoni*
	2010	M	Ascarid, *Ortleppascaris antipini*
	2010	N	*Dujardinascaris waltoni*
	2010	O	Ascarid, *Dujardinascaris waltoni*, *Goezia* sp.
	2010	S	*Dujardinascaris waltoni*
	2010	T	Ascarid, *Brevimulticaecum tenuicolle*, *Dujardinascaris waltoni*, *Ortleppascaris antipini*
	2010	Z	*Dujardinascaris waltoni*
	2011	1	Ascarid, *Acanthostomum pavidum*, *Proctocaecum coronarium*, *Sebekia mississippiensis*
	2011	2	Ascarid, *Brevimulticaecum tenuicolle*, *Dujardinascaris waltoni*, *Eustronglyides* sp., *Ortleppascaris antipini*
	2011	3	*Acanthostomum pavidum*, *Proctocaecum coronarium*, *Sebekia mississippiensis*
	2011	4	Ascarid, *Acanthostomum pavidum*, *Proctocaecum coronarium*, *Timoniella loosi*
	2011	6	*Dujardinascaris waltoni*
	2011	11	Ascarid, *Dujardinascaris waltoni*, *Ortleppascaris antipini*, *Terranova lanceolata*
	2011	15	*Sebekia mississippiensis*
	2011	16	*Dujardinascaris waltoni*
	2011	18	*Sebekia mississippiensis*
	2011	19	*Dujardinascaris waltoni*, *Sebekia mississippiensis*
	2011	20	*Sebekia mississippiensis*
	2011	P	*Brevimulticaecum tenuicolle*, *Ortleppascaris antipini*
	2011	T	Ascarid, *Brevimulticaecum tenuicolle*, *Dujardinascaris waltoni*, *Ortleppascaris antipini*
	2011	U	*Dujardinascaris waltoni*, *Sebekia mississippiensis*, *Terranova lanceolata*
	2012	H	*Dujardinascaris waltoni*
	2012	J	Ascarid, *Brevimulticaecum tenuicolle*, *Dujardinascaris waltoni*, *Ortleppascaris antipini*
SWLA	2009	B	Ascarid, *Brevimulticaecum tenuicolle*, *Ortleppascaris antipini*
	2009	F	Ascarid, *Dujardinascaris waltoni*, *Ortleppascaris antipini*
	2010	2	Ascarid, *Brevimulticaecum tenuicolle*, *Ortleppascaris antipini*, *Terranova lanceolata*
	2010	4	*Dujardinascaris waltoni*
	2010	8	*Dujardinascaris waltoni*
	2010	12	*Dujardinascaris waltoni*
	2010	13	Ascarid, *Brevimulticaecum tenuicolle*, *Dujardinascaris waltoni*, *Ortleppascaris antipini*
	2010	15	*Dujardinascaris waltoni*
	2010	50	*Dujardinascaris waltoni*
	2011	C	*Sebekia mississippiensis*
	2011	D	*Sebekia mississippiensis*
	2011	E	*Sebekia mississippiensis*
	2011	F	*Sebekia mississippiensis*
	2011	H	*Dujardinascaris waltoni*
	2011	I	Ascarid, *Brevimulticaecum tenuicolle*, *Acanthostomum pavidum*, *Archaeodiplostomum acetubulata*, *Polycotyle ornata*, *Pseudocrocodilicola georgiana*, *Dujardinascaris waltoni*, *Ortleppascaris antipini*, *Proctocaecum coronarium*
	2011	K	Ascarid, *Brevimulticaecum tenuicolle*, *Dujardinascaris waltoni*, *Ortleppascaris antipini*, *Sebekia mississippiensis*
	2011	L	Ascarid, *Acanthostomum pavidum*, *Archaeodiplostomum acetubulata*, *Proctocaecum coronarium*, *Pseudocrocodilicola americana*, *Sebekia mississippiensis*
	2011	N	*Sebekia mississippiensis*
	2011	0	*Sebekia mississippiensis*
	2011	Y	*Ortleppascaris antipini*
	2011	2	Ascarid, *Brevimulticaecum tenuicolle*, *Dujardinascaris waltoni*, *Eustronglyides* sp., *Ortleppascaris antipini*
	2011	5	Ascarid, *Dujardinascaris waltoni*, *Ortleppascaris antipini*, *Sebekia mississippiensis*
	2011	6	*Dujardinascaris waltoni*
	2011	7	*Sebekia mississippiensis*
	2011	10	*Sebekia mississippiensis*
	2011	17	*Sebekia mississippiensis*
	2011	N	*Sebekia mississippiensis*
FL	2011	A	*Dujardinascaris waltoni*, *Brevimulticaecum tenuicolle*, *Ortleppascaris antipini*, *Sebekia mississippiensis*
	2011	B	*Dujardinascaris waltoni*, *Brevimulticaecum tenuicolle*, *Ortleppascaris antipini*
	2011	E	*Dujardinascaris waltoni*, *Ortleppascaris antipini*, *Sebekia mississippiensis*
	2011	F	Ascarid, *Dujardinascaris waltoni*, *Ortleppascaris antipini*, *Sebekia mississippiensis*
	2011	G	*Sebekia mississippiensis*
	2011	H	*Brevimulticaecum tenuicolle*, *Dujardinascaris waltoni*, *Eustronglyides* sp., *Ortleppascaris antipini*, *Sebekia mississippiensis*
	2011	I	*Sebekia mississippiensis*
	2011	J	*Dujardinascaris waltoni*, *Eustronglyides* sp., *Sebekia mississippiensis*
	2011	K	*Sebekia mississippiensis*
	2011	L	*Sebekia mississippiensis*
	2011	M	*Dujardinascaris waltoni*, *Sebekia mississippiensis*
	2011	N	Ascarid, *Brevimulticaecum tenuicolle*, *Dujardinascaris waltoni*, *Ortleppascaris antipini*, *Sebekia mississippiensis*
	2011	O	*Brevimulticaecum tenuicolle*, *Dujardinascaris waltoni*, *Eustronglyides* sp., *Goezia* sp., *Ortleppascaris antipini*, *Sebekia mississippiensis*
	2011	Q	*Sebekia mississippiensis*
	2011	R	*Sebekia mississippiensis*
	2011	S	*Dujardinascaris waltoni*, *Sebekia mississippiensis*
	2011	T	*Sebekia mississippiensis*
	2012	A	*Brevimulticaecum tenuicolle*, *Dujardinascaris waltoni*, *Ortleppascaris antipini*
	2012	B	Ascarid, *Brevimulticaecum tenuicolle*, *Dujardinascaris waltoni*, *Eustronglyides* sp., *Goezia* sp., *Ortleppascaris antipini*
	2012	C	Ascarid, *Dujardinascaris waltoni*, *Ortleppascaris antipini*
	2012	D	*Dujardinascaris waltoni*, *Ortleppascaris antipini*
	2012	E	*Dujardinascaris waltoni*
	2012	G	*Dujardinascaris waltoni*, *Ortleppascaris antipini*
	2012	H	Ascarid, *Brevimulticaecum tenuicolle*, *Dujardinascaris waltoni*, *Ortleppascaris antipini*
	2012	I	Ascarid, *Dujardinascaris waltoni*, *Ortleppascaris antipini*
	2012	J	*Dujardinascaris waltoni*

* Numbers and letters under column “Host” identifies the tag of each alligator individual, i.e., “Alligator 13 2009,” Alligator A 2009”, etc.

A total of 106 alligators (i.e. livers) from 2010–2012 were used in our study. Samples of livers from geographic locations were collected as follows: SWLA (n = 40) and SELA (n = 39) during the 2010–2012 annual Louisiana alligator harvest, and FL (n = 27) during the 2011 and 2012 FL alligator harvests. S2 Table lists number of alligators from each region and site per year. Livers were chosen for the analysis of heavy metals because heavy metals are concentrated in the liver due to its high blood supply and function of detoxification [34–37]. Liver samples were stored in a freezer at -80°C. Liver samples and parasites samples were eventually transported to McNeese University for heavy metal analysis.

All sampling procedures were approved by the LDWF and FFW. UCLA IACUC permission or approval was not required as the animals collected were sacrificed by the time of our sample collection because of a legal, statewide animal harvest.

### ICP-OES Analysis

In order to prepare samples for heavy metal analysis, liver samples collected from alligators, were dried overnight at 115°C and the mass measured to the nearest 0.01 g (S3 Table). Parasite specimens from each host and bio-location were dried in the same manner, and mass measured to the nearest 0.001 g (S3 Table). Parasite samples were categorized as lung pentastomids, stomach nematodes, intestinal trematodes, and intestinal nematodes. Multiple parasite species were found within the stomach and intestines, however it was predicted the low quantitative representation of each species would not result in metal levels above detection limits if analyzed for heavy metals separately. Therefore, parasites were pooled into the appropriate parasitic bio-location category and analyzed as one sample. Dry samples were ashed for 10–12 hours at 500°C in a KSL-1100x muffle furnace (MTI Corp, Richmond, CA, USA) to allow the samples to cool. Samples were then digested with 7 ml of 10% analytical grade nitric acid (Sigma-Aldrich, St. Louis, MO, USA), and poured into a 10 ml volumetric flask. The flasks were filled to volume with dionized water, and then transferred to 15ml polypropylene centrifuge tubes for analysis. The preparation and digestion process of samples for heavy metal analysis are further described elsewhere [38,39].

Metals examined for this study were As, Cd, Cu, Fe, Pb, Se, and Zn. All samples were analyzed for metals using a Varian 715 ES inductively coupled plasma atomic emission spectroscopy (ICP-OES) instrument at McNeese State University. The emission wavelengths used for the analyses of As, Cd, Cu, Fe, Pb, Se, and Zn were 188.980, 214.439, 327.395, 259.94, 220.353, 196.026, and 213.857 nm, respectively. A standard solution containing 5 ppm of all analytes was analyzed after every 30 samples to check for instrumental drift. Calibrations of ICP-OES were achieved using five standards (0, 1, 2, 5, 10, and 20 ppm). The data collected from the ICP instrument, expressed in mg/L solution, was multiplied by the volume of the sample, expressed in L. The resulting mass of metal in the sample, expressed in mg, was divided by the dry weight of the sample, expressed in kg, resulting in mg of metal/kg of dried sample, or ppm.

### Statistical Analyses

To determine if alligators and parasites accumulated heavy metals at different concentrations, we first performed a Wilcoxon-Signed Rank test to independently analyze the variation of Fe, As, Cd, Cu, Pb, Se, and Zn between alligators and parasites comprehensively. We then performed a second Wilcoxon-Signed Rank test in which we analyzed the accumulation of heavy metals independently between alligator tissue and each parasite category (i.e., lung pentastomids, stomach nematodes, intestinal trematodes, and intestinal nematodes).

We also performed several Kruskal-Wallis Rank Sum tests to analyze heavy metal variation between an individual host and its parasites. First, we examined the variation of metal bioaccumulation between parasites of the four parasite categories. Secondly, we examined the difference of heavy metal levels between the four designated groups of parasites, in addition to alligators, among the geographic locations (i.e., Florida (FL), Louisiana East Zone (SELA) and Louisiana West Zone (SWLA)). Besides examining the variation of bioaccumulation among geographic locations, this statistical analysis also helped us to examine if there was any statistical inconsistency of metal bioaccumulation between alligators and their parasites from the same locality.

Finally, we used a Spearman rank correlation to determine if there was a relationship between heavy metal levels and parasite abundance. Spearman rank correlations, Kruskal-Wallis and Wilcoxon-Signed Rank tests were performed with program R [40]. False discovery rates (FDRs) were calculated for each group of statistical analyses to examine Type I error of multiple comparisons [41,42]. FDRs illustrate the projected percent of false predictions in a set of predictions. For example, an FDR of 0.15 states that 15% of predictions in an analysis are false, and 85% of predictions are correct. The lower FDR strengthens the confidence level for p-values or predictions. All statistical tests were considered statistically significantly different when p ≤ 0.05.

## Results

Overall, the individual accumulation of As, Cu, Se, and Zn were significantly higher in parasites collectively (As, Cu, Se, and Zn, Wilcoxon-Signed Rank tests = p < 0.0001, FDRs < 0.001), whereas Fe, and Pb were each significantly higher in alligators when examined conjointly (Fe, and Pb, Wilcoxon-Signed Rank tests = p < 0.0001, FDRs < 0.001) (Table 2). Yet, Fe levels were significantly higher in intestinal trematodes than alligators when analyzed exclusively from other parasitic groups (Wilcoxon-Signed Rank test = 191, p = 0.02617).

**Table 2:** Average heavy metal concentrations (mg/kg) in alligators and parasites (mean ± standard deviations).

	As	Cd	Cu	Fe	Pb	Se	Zn
Alligator	0.8 ± 6.0	0.1 ± 0.3	39.2 ± 125.6	5144 ± 23320.3	4.3 ± 10.0	< 0.1 ± 0.9	87.1 ± 268.5
Parasites	62.1 ± 156.3	0.1 ± 0.9	153.2 ± 283.4	1012.4 ± 2168.7	0.7 ± 4.3	19.5 ± 137.2	1213.6 ± 3361.5

### Variation of Heavy Metal Concentrations among Parasite Groups

Table 3 illustrates average heavy metal accumulation among parasite groups, and Table 4 summarizes specific statistical heavy metal variation among parasite groups. In general, independent analyses of As, Cu, Fe, Se, and Zn showed significantly higher accumulation among intestinal trematodes relative to other parasites (As, Cu, Fe, Se, and Zn, Kruskal Wallis chi-squared tests, df = 3, p < 0.001, FDRs < 0.089). Stomach nematodes had significantly higher accumulation of Pb compared to other parasite categories (Kruskal-Wallis chi-squared = 10.367, df = 3, p = 0.035, FDR = 0.068). Individual analysis of Cd among parasite groups did not significantly vary (Cd, Kruskal Wallis chi-squared tests, p > 0.05).

**Table 3:** Average heavy metal concentrations (mg/kg ± standard deviation) for different parasite groups.

	As	Cd	Cu	Fe	Pb	Se	Zn
**Lung Pentastomids**	88.9 ± 134.9	0	120.5 ± 118.2	955.6 ± 1026.0	0	39.9 ± 119.3	1737.5 ± 3541.8
**Stomach Nematodes**	7.0 ± 20.5	0.2 ± 0.1	58.0 ± 97.2	461.0 ± 1166.4	1.7 ± 6.6	< 0.1 ± 96.8	802.9 ± 3836.4
**Intestinal Trematodes**	232.6 ± 289.9	1.6 ± 4.0	960.2 ± 663.1	5339.7 ± 4256.3	0	115.4 ± 167.0	1590.4 ± 1012.6
**Intestinal Nematodes**	81.6 ± 237.2	0.1 ± 0.3	184.2 ± 241.7	412.5 ± 382,8	0	5.0 ± 132.2	1103 ± 2252.3

**Table 4:** Summary of statistically significant heavy metal variation among pentastomids, trematodes and nematodes. Parasites with significantly higher levels of heavy metal concentrations are marked with (*). Statistical analyses were performed with Wilcoxon-Signed Rank tests. FDRs are provided to show the projected percent of false predictions in the set of predictions.

Statistical Analyses Between Parasites	Heavy Metals	p-value	FDR
Intestinal Trematodes * vs. Lung Pentastomids	As, Cu, and Fe	As: p = 0.04; Cu: p < 0.001; Fe: p < 0.001	As: 5.4e-2; Cu: 1e-5; Fe: 1.4e-5
Intestinal Trematodes* vs. Intestinal Nematodes	As, Cu, and Fe	As: p = 0.009; Cu: p = 0.002; Fe: p < 0.001	As: 1.9e-2; Cu: 2e-3; Fe: 4.5e-6
Intestinal Trematodes * vs. Stomach Nematodes	As, Cu, Fe, Se, and Zn	As: p < 0.001; Cu: p < 0.001; Fe: p < 0.001; Se: p = 0.05; Zn: p < 0.001	As: 2.6e-6; Cu: 9.3e-7; Fe: 1e-5; Se: 0.07; Zn: 1.5e-4
Intestinal Nematodes* vs. Stomach Nematodes	Cu, Fe, and Zn	Cu: p < 0.001; Fe: p = 0.03; Zn: p = 0.002	Cu: 1.3e-3; Fe: 3.3e-2; Zn: 3.3–3
Stomach Nematodes* vs. Lung Pentastomids	As	As: p < 0.001	As: 1.7e-6
Lung Pentastomids* vs. Intestinal Nematodes	As, Fe, and Zn	As: p = 0.02; Fe: p < 0.001; Zn: p = 0.03	As: 3.5e-2; Fe: 1.4e-3; Zn: 3.3e-2

### Variation of Heavy Metal Accumulation among Locations

Although As, Cu, Fe and Zn levels did not significantly differ among alligators from SWLA, SELA, or FL (Kruskal Wallis chi-squared, df = 2, p > 0.05), concentrations of these trace metals individually significantly varied among parasites collectively (As, Cu, Fe, and Zn, Kruskal-Wallis chi-squared, df = 2, p < 0.001, FDRs < 0.001) (Table 5). The highest levels of As, Fe, Cu, and Zn were found among parasites from SWLA. In contrast, the respective levels of Cd, Pb, and Se did not statistically vary among parasites throughout locations (Cd, Pb, and Se, Kruskal-Wallis chi-squared tests, df = 2, p > 0.05). Analogous to these results, individual levels of Cd, Pb, and Se did not vary among alligators from the three locations (Kruskal-Wallis chi-squared tests, df = 2, p > 0.05).

**Table 5:** Comparison between geographic zones of mean (± standard deviation) heavy metal concentrations (mg/kg) from alligators and their parasites.

	As	Cd	Cu	Fe	Pb	Se	Zn
**SELA**							
Alligator	0.2 ± 0.3	0.1 ± 0.1	25.1 ± 24.9	2507.9 ± 2469.6	4.1 ± 10.3	< 0.1 ± 0.3	60.3 ± 25.4
Parasites	31.7 ± 77.0	0.1 ± 0.2	160.6 ± 239.6	1273.6 ± 2018.0	0.2 ± 0.8	32.6 ± 0.8	1901.4 ± 5368.4
**SWLA**							
Alligator	0.2 ± 0.2	0.1 ± 0.2	31.4 ± 20.1	3876.6 ± 3733.0	4.7 ± 10.0	< 0.1 ± 0.6	68.6 ± 22.7
Parasites	149.9 ± 245.2	0.3 ± 1.7	313.0 ± 405.2	1568.2 ± 3257.2	1.3 ± 7.3	17.9 ± 233.1	2003.8 ± 3249.3
**FL**							
Alligator	2.5 ± 12.1	0.22 ± 0.6	75.3 ± 250.4	11677.1 ± 46816.19	4.2 ± 10.0	0.2 ± 1.6	159.9 ± 541.2
Parasites	15.7 ± 43.4	0.02 ± 0.1	30.7 ± 38.9	442.7 ± 593.1	0.6 ± 2.11	12.7 ± 38.1	210.8 ± 197.2

In addition to analyzing the variation of heavy metal bioaccumulation among the geographic locations of parasites en masse, we also statistically examined the variation of As, Cu, Cd, Fe, Pb, Se, and Zn levels in each parasite category among geographic locations. Table 6 summarizes our statistical results of heavy metal variation among parasites and alligators throughout geographic regions. In general, the accumulation of heavy metals shows an overall pattern of higher levels among SWLA parasites, except for pentastomids. Interestingly, even though As accumulation was not statistically higher among SELA alligators, our data shows bioaccumulation of As among pentastomids was significantly higher in SELA in comparison to SWLA and FL (Kruskal-Wallis chi-squared = 6.239, df = 2, p = 0.044, FDR = 0.1). We found no statistical difference of As, Cu, Cd, Fe, Pb, Se, and Zn among intestinal trematodes throughout the regions (all p > 0.05).

**Table 6:** Statistical comparison by region (SELA, SWLA, and FL) of heavy metals from different parasite groups. Statistical analyses were performed with Kruskal-Wallis chi-squared tests; region highlighted in table had significantly higher concentrations compared to other areas.

Parasite Category	Region	Heavy Metals	p value	FDR
Trematodes	–	–	all p > 0.05	–
Intestinal Nematodes	SWLA	Cu, Zn	Cu: p < 0.002; Zn: p < 0.001	Cu: 0.008; Zn: 0.007
Stomach Nematodes	SWLA	Cu, Fe, and Zn	Cu: p = 0.01; Fe: p = 0.03; Zn: p = 0.002	Cu: 0.05; Fe: 0.08; Zn: 0.02
Lung Pentastomids	SELA and SWLA	As^a^, Cu^b^, Zn^b^	As: p = 0.04; Cu: p = 0.08; Zn: p < 0.001	As: 0.1; Cu: 0.03; Zn: 0.004

^a^ Heavy metal bioaccumulation statistically higher in SELA

^b^ Heavy metal bioaccumulation statistically higher in SWLA

### Correlation of Parasite Abundance to Heavy Metal Concentrations

Parasite abundance did not show any significant relationship to Cd, Cu, Fe, Pb, Se, and Zn levels (Cd, Cu, Fe, Pb, Se, and Zn, Spearman rank correlations, p > 0.05). However, the increase of parasitic abundance was found to significantly correlate to lower levels of As among alligators (S = 264943.6, p = 0.042, r_s_ = -0.194, FDR = 0.15).

## Discussion

Successive predation through the food web allows for organic and inorganic elements to accumulate and increase in concentration within organismal tissue, particularly among higher trophic levels [22,37,43]. Hence, top-level predators generally possess higher concentration levels of heavy metals in comparison to lower trophic organisms [37,44–46]. In this study, we analyzed the variation of heavy metal bioaccumulation among the American alligator and its parasites, and assessed the effect of heavy metals on alligator parasitism via parasitic abundance. Additionally, we evaluated the role of alligator parasites as a possible important determinant in the detection of cryptic toxic levels of heavy metals within alligators, and in the environment.

### Heavy Metal Variation among Alligators and Parasites

Consumed prey of alligators is likely the principal source of heavy metals detected among both alligators and their parasites. Despite this common source, our data shows that parasites and alligators bioaccumulate heavy metals at different rates. For example, parasites accumulate As, Cu, Se, and Zn at higher concentrations than their hosts, whereas alligators show greater tendency to accumulate Fe, Cd, and Pb relative to parasites. We did find a few circumstances of Fe and Pb bioaccumulation greater among parasites (i.e., trematodes and stomach nematodes), however, the overall trend showed lower levels of these particular metals among parasites collectively in comparison to alligators. Based on these data (and relative to abiotic and biotic samples shortly to be discussed), we suggest the use of alligators or their parasites as biological indicators may be dependent on the particular trace metal to be examined. For example, the use of alligator parasites in the detection of Zn levels in the environment would be more advantageous than its reptilian host. The mean Zn concentration level from alligators collectively in our study was 87 mg/kg. In contrast, mean bioaccumulation of Zn in parasites was 1213 mg/kg. Intestinal parasites, which were found to accumulate Zn at much higher levels than pentastomids and gastric nematodes in our study, showed a mean of 1530.2 mg/kg. To further corroborate the above argument, levels of Zn measured from crawfish samples [47] near our collection localities in Vermillion Parish in SWLA during the 2009 harvest had lower levels of Zn in comparison to intestinal parasites (0.05 mg/kg vs. 552.54 mg/kg). Additionally, levels of Cu were higher among alligator intestinal parasites than crawfish 103.6 mg/kg vs. 0.03 mg/kg). Overall, these data are consistent with previous studies that show Zn and Cu uptake in parasites are higher when compared to the tissues of invertebrates, or organisms of lower trophic levels [12,19,18,22,24,25].

Our data is unlike previous research concerning metal accumulation in host-parasite systems (see references [11,12,21,22]), as alligator parasites did not biomagnify all trace metals at levels of magnitude higher than its host. Thus, what biological or physiological feature among alligators or their parasites causes this variation of heavy metal bioaccumulation that contradicts findings from previous host-parasite systems? Perhaps the higher concentrations of Fe, Cd, and Pb among alligators is a consequence of increase binding rates to metallothioneins in comparison to As, Cu, Se, and Zn. Metallothioneins are important hepatic proteins that bind to heavy metals, and assist in the process of detoxification [48–50]. Alternatively, As, Cu, Se, and Zn may be more readily absorbed across the tegument of parasites than other trace metals, allowing Fe, Cd, and Pb to circulate within the alligator circulatory system at higher concentrations. However, these are only speculations as our study is purely descriptive, and we did not concentrate on the biomechanics of heavy metal absorption between alligators and their parasites. Future experiments analyzing the rate of metallothioneins binding to various heavy metals in alligators should be conducted, as these data could provide beneficial information that may help clarify the variation of heavy metal accumulation between alligators and their parasites.

### Variation of Heavy Metals among Parasites

A particular pattern arose during our analysis of heavy metal differentiation among the different type of parasites. First, stomach nematodes do not accumulate heavy metals, except Pb, in high concentrations. Lead objects, such as bullet casings (author pers. obs.), ingested by alligators may, in part, contribute to the high lead accumulation found among stomach nematodes. Lead casings are not easily digested, thus can accrue within the stomach over time. Secondly, lung pentastomids accumulated heavy metals at higher concentrations than intestinal nematodes, yet the accumulation levels of metals were overall greater among intestinal trematodes relative to both pentastomids and intestinal nematodes. Taken collectively, nematodes seem to be poor bioindicators of trace metal concentrations within the environment and host. Intestinal trematodes, however, appear to be exceptional candidates as biological indicators of heavy metals.

It is likely trematode biology, life history, and occupation of biological niche space greatly affects their ability to accumulate metals to high concentrations. The epithelium of nematodes and pentastomids is non-cellular and made up of collagen or chitin [51]. Nutrient uptake is non-passive, in which parasites feed on ingested food, or on the blood or lymph of its host [51]. In contrast, the epithelium of trematodes is made of cells, which are absorptive by nature [51]. Some nutrients are thus absorbed through the digestion of blood or host tissue since trematodes imbed into the tissue of their host [51]. Intestinal alligator parasites attach along the intestinal tract, mainly occupying luminal space within the small intestines (author’s pers. obs). The small intestine is the primary site in which nutrients, as well as heavy metals, are absorbed into the blood stream across the mucosa of vertebrates [52–54]. Based on this information, intestinal trematodes are likely accumulating metals prior to their circulation within the alligator blood stream. This could explain the high accumulation of Fe among intestinal trematodes compared to alligators, particularly since Fe concentrates at high levels in vertebrate liver [55]. Conclusively, the primary access to a majority of heavy metals by intestinal trematodes likely results in their higher heavy metal accumulation levels compared to other alligator parasitic taxa.

### Variation of Heavy Metals among Geographic Locations

**Fig 2:**
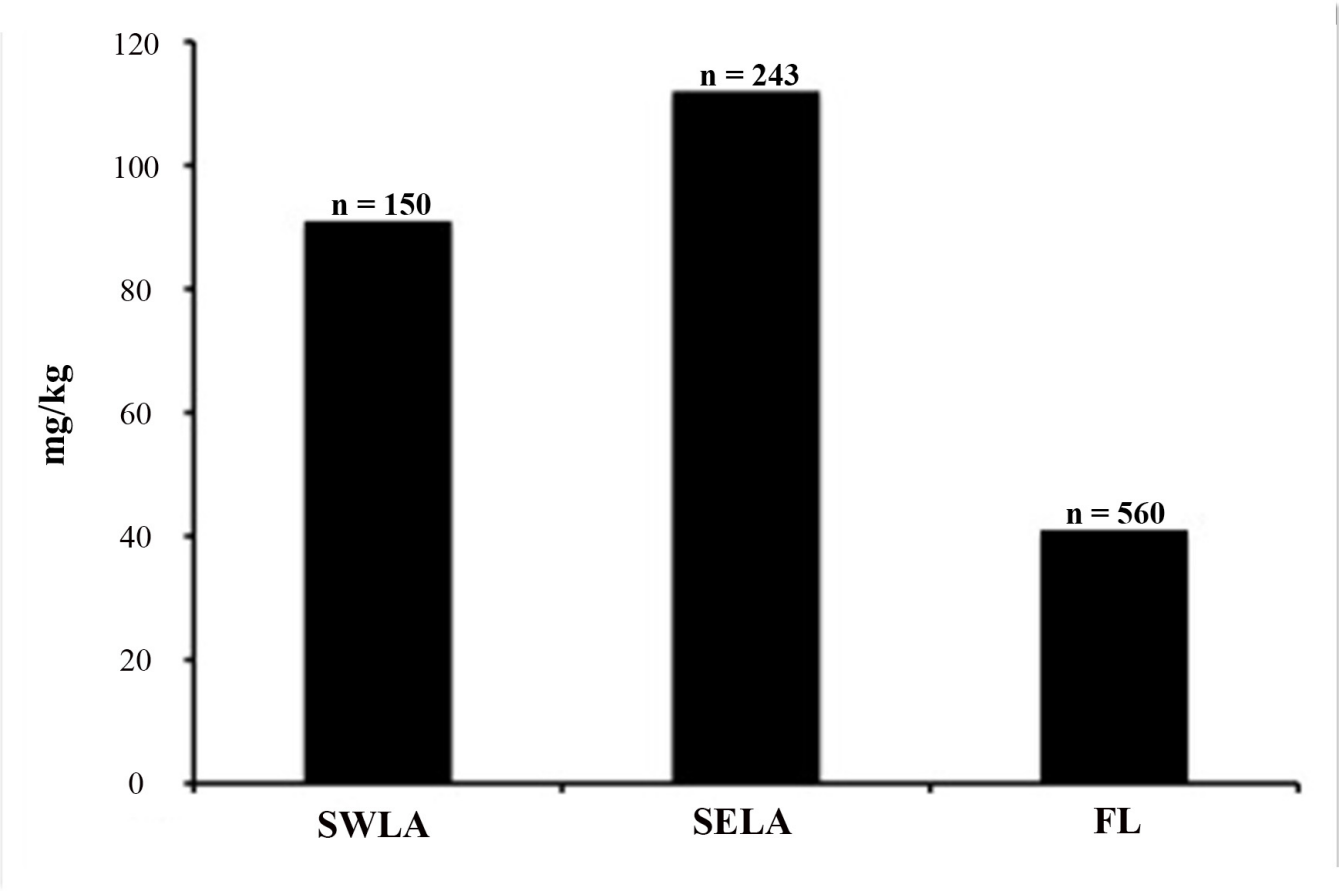
Comparative data of As concentrations among pentastomids from SWLA, SELA, and FL. Quantity of pentastomids analyzed from each region is shown (n =).

Given the diverse array of microhabitats throughout southern Louisiana and north-central Florida in which alligator and parasite samples were collected, it was anticipated that heavy metal levels among host and parasites would differ as a reflection of the different environments and prey species throughout the range. However, bioaccumulation levels of heavy metals did not differ significantly among locality sites. However, en masse, parasites showed higher accumulation of As, Fe, Cu, and Zn in SWLA. Interestingly, Fe, Cu, and Zn coincide with the high levels detected from their alligator hosts in SWLA or Florida. The high bioaccumulation of As among parasites, however, did not correlate. Collectively as a group, parasites from SWLA concentrated As at a higher magnitude (5350 mg/kg vs. 7 mg/kg detected among SWLA alligators), however levels of As were slightly higher among alligators from SELA (11 mg/kg). Yet, despite these contrasting results, the bioaccumulation of As was highest among lung pentastomids from SELA (Fig 2). The above results generate two plausible explanations. First, it is hypothesized that a large disparity between parasite and host bioaccumulation levels (i.e., a high ratio [Cparasite/Chost]) are indicative of acute pollutant exposure, whereas long or chronic exposure of the pollutant correlates with high concentrations in both the host and parasites [17,19,22]. Thus, the significant difference of As bioaccumulation between alligators and their collective parasites illustrates the possible acute exposure to this trace metal within SWLA (Table 7). Secondly, given that lungs are the site of infection for pentastomids, the biomagnification of As among SELA pentastomids is perhaps caused by indirect (excess circulation of As among alligator hosts via consumed prey), and direct (airborne, inhaled through alligator respiratory tract) exposure. Compared to other collection sites, SELA is highly industrialized and urbanized, which can contribute to higher soil, water, and aerial As pollution [56–58]. Based on these data, pentastomids, in addition to trematodes, may be ideal bioindicators of environmental and alligator accumulation of As.

**Table 7:** Ratio of bioaccumulation levels (Cparasite/Chost) between alligators and their parasites collectively among geographic zones. High ratios are indicative of acute exposure to heavy metals, and low ratios are indicative of chronic exposure to heavy metal pollution.

	As	Cd	Cu	Fe	Pb	Se	Zn
**FL**	10.74	0.16	0.69	0.06	0.24	97.75	2.25
**SELA**	56.7	0.52	3.03	0.24	0.03	647.63	16.5
**SWLA**	796.77	2.48	11.16	0.46	0.3	333.79	29.91

### Correlation of Parasite Abundance to As

Recent investigations show elevated heavy metal concentrations within the environment can decrease the population size of parasites by shortening their longevity, impeding life cycle transmissions, or negatively affecting intermediate host abundance, which can directly affect parasite abundance and intensity within a host population [12,14,59]. The magnitude of As concentrations seem to play a crucial role in the abundance of parasites. This contrasts other host-parasite systems, which show no relation between parasite abundance and the degree of As accumulation [12]. Our results, however, suggest parasite abundance decreases in accordance with higher levels of As as we found a negative correlation between parasite abundance and As concentration. The direct cause for the decline of the parasite infracommunity is unknown as there is insufficient data of the physiological consequences of As exposure in parasites. Yet, perhaps the adverse effects of As towards parasites is similar to those in free-living organisms. Among vertebrates and macroinvertebrates, toxic levels of As are associated with declines in immunity, neurological and vascular complications, and a decrease in growth or maturation [60–63]. Organisms can become exposed to As through air, food, or water [60–63]. Given the detrimental effects of As in free-living organisms, perhaps toxic levels of As can alter the physiological behavior of free-living stages of parasites, thus disrupting the successful transmission to intermediate hosts. Alternatively, toxic levels of As may interfere with the ontogenetic growth of parasites, similar to free-living organisms.

### Can Alligator Parasites Be Buffers of Alligator Heavy Metal Toxicity, and Biological Indicators of Environmental Pollution?

In the presence of environmental pollution, the phenomenon of parasites employed as a biological buffer against heavy metal toxicity within the host is a conceivable concept. For instance, minks from the Great Basin Lakes region in Canada infected by lung parasites illustrated lower concentration levels of heavy metals relative to uninfected minks [64]. It is also probable that parasites only absorb particular heavy metals into their tissue. Several heavy metals (Cd, Cr, Cu, Fe, Mn, Ni, Pb, and Zn) were analyzed among red foxes, yet only infected foxes with *Echinococcus multilocularis* illustrated lower levels of Cd and Pb compared to uninfected red foxes [65]. In conjunction with our study, these investigations suggest parasites capability to bioaccumulate particular heavy metals at higher concentrations than their host perhaps delays, or hinders, the onset of heavy metal toxicity. This is particularly advantageous for organisms of higher trophic levels, as certain elements are known to concentrate at higher levels towards the top of the food web [37,44–46,66,67]. It should also be considered that this biological buffering system would mask the levels of environmental contamination to which alligators are exposed, which would cause lower levels detected within the host. Subsequently, heavy metal analyses solely among host tissue would thus provide inaccurate results of environmental pollution. Relative to our data, we show plausible evidence of the possible buffering role of alligator parasites as the bioaccumulation of heavy metals among parasites was generally higher in comparison to their archaic reptilian host. In particular, our data suggests parasites may buffer alligators from the toxic effects of As, Cu, Se, and Zn. This data is similar to the study of *E*. *multilocularis* and red foxes, suggesting that parasites may better absorb or buffer their hosts from particular heavy metals analogous to other heavy metals [65]. However, it must be taken into consideration that extreme levels of these metals could likely cause parasite mortality. Yet, whether rising environmental contamination causes increase metal bioaccumulation or mortality of parasites, either outcome strengthens the applicability of parasites as biological indicators [18,19].

**Fig 3:**
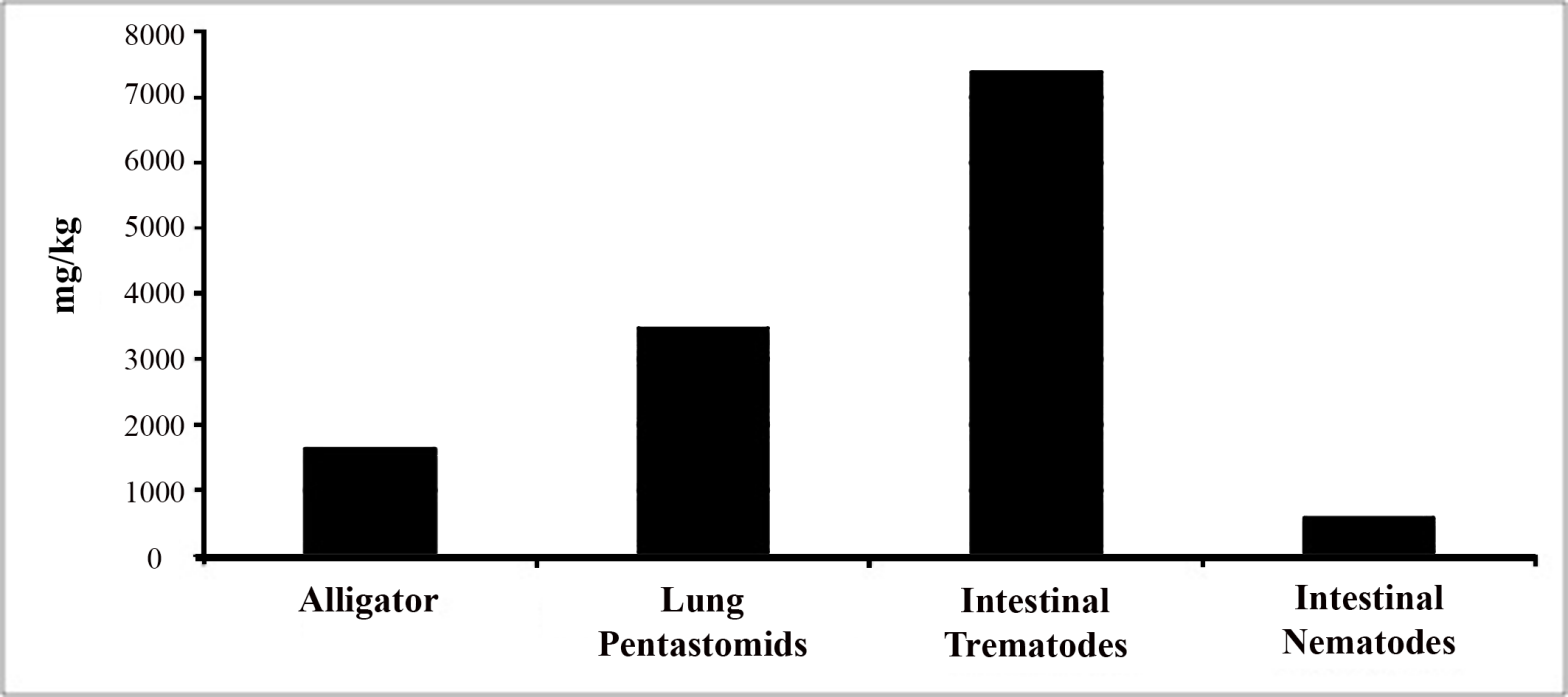
Comparative data of the total heavy metal concentration from an individual alligator from Iberville, Louisiana from the 2011 SELA alligator harvest.

**Fig 4:**
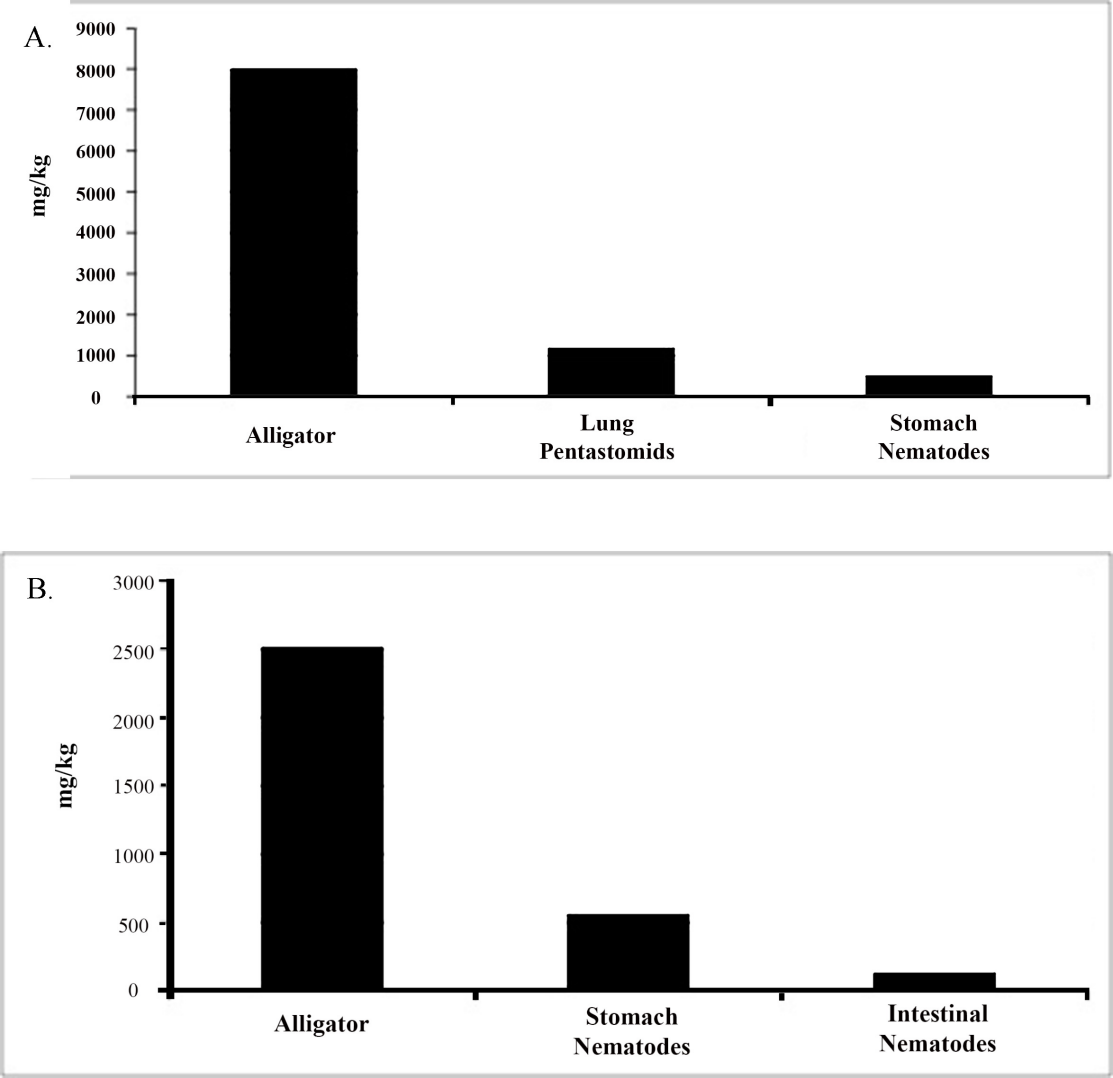
Comparative data of the total heavy metal concentration from Lake Loochloosa, FL during (a) 2011, and (b) 2012 alligator harvests. Each graph represents an individual collected from that year selected at random.

Amid the previous heavy metal studies of fish-parasite systems, the accumulation and response of parasites to environmental pollution is relatively rapid in comparison to its host [19]. Our data showed many examples of high metal bioaccumulations in parasites, particularly among intestinal trematodes, in comparison to alligators. For instance, comparative data from Iberville, Louisiana (SELA) during the 2011 harvest illustrated the combined levels of heavy metals were very high among trematodes, whereas the collective accumulation of heavy metals among hosts were low (Fig 3). In agreement with previous theory [19], this example may reflect acute discharge of heavy metals into the environment. In contrast, alligators from Lake Loochloosa, FL were found to have higher heavy metal accumulation relative to their parasites in 2011 and 2012 (Fig 4). Accordingly, this data may reflect the overall low or chronic release of these metals into the environment. Hence, the comparison of metal accumulation between parasites and alligators can potentially reveal cryptic data about the acute and chronic chemical state of the environment, which can help establish the necessary management to counteract environmental pollution.

To further determine the role of alligators and their parasites as biological indicators of their environment, we compared data from previous studies that performed heavy metal analyses in some of our sampling area on soil and crawfish [47,68]. In general, intestinal nematodes of alligators collected from Rockefeller Wildlife Refuge during the 2009 annual alligator harvest in SWLA revealed higher concentrations of Cu and Zn than levels detected in soil (intestinal nematodes: Cu = 389 mg/kg, Zn = 398.2 mg/kg vs. soil: Cu = 28 mg/kg, Zn = 72 mg/kg) [68]. Additionally, bioaccumulation of Cu and Zn in western Louisiana in 2010 were found to be higher among parasites (Cu = 571.8 mg/kg; Zn = 5033.4 mg/kg) than both alligators (Cu = 5.5 mg/kg; Zn = 54.6 mg/kg) and crawfish (Cu = 34.9 mg/kg; Zn = 47.3 mg/kg) [47]. These results are similar to previous studies comparing heavy metal accumulation between hosts and parasites [17,22,24]. For example, Cu and Zn levels were present at significantly higher concentration levels among acanthocephalans than its zebra mussel hosts [22]. Concomitantly, Fe also shows trends of higher accumulation among parasites than its host. *Pomphorhynchus laevis*, a parasite of the barbell *Barbus barbus*, accumulated Fe better than its host as well another organism from the study site, *Dreissena polymorpha* [24]. In contrast, the bioaccumulation of Fe among alligators was at degrees of magnitude higher than both crawfish and parasites [47]. Relative to the two above examples, our results generate an argument for the use of alligators and their parasites as bioindicators for heavy metals.

Previous studies have suggested crocodilians accumulate heavy metals at low rates, or are tolerant to high levels of pollution [1]. However, it is possible for high levels or symptoms of heavy metal toxicity to be delayed or masked [1,28]. Based on our data, and the likely buffering mechanism of alligator parasites, we suggest that future investigations of heavy metal toxicity in crocodilians should examine heavy metal levels in their parasites, particularly intestinal trematodes. As previously stated, our findings show intestinal trematodes are likely superior biomagnifiers of heavy metal toxicity among crocodilians and their environment relative to other alligator parasites. We anticipate this data, together with other results in this study, emphasize the beneficial use of alligator parasites as indicators of enigmatic heavy metal concentrations in crocodilians and their environment, as well as generate a new perspective about the role parasites may have in the tolerability of heavy metals among crocodilians. Further investigations on the ecological and biological intake of heavy metals by alligators and their parasites should improve monitoring of environmental pollution, which can further implement the provisions essential for the sustainability of crocodilians and their habitats.

## Supporting Information

S1 Table Geographic locations of collection sites in East Louisiana, West Louisiana, and Florida. (DOC)

S2 Table Summary of alligators from each region and site per year. (DOC)

S3 Table Dry weight (g) of alligator liver samples, and parasite specimens from each year and geographic zone as organized in heavy metal analysis. Samples 30 and 60 in each Tray are identified as Blanks as these were a standard solution containing 5 ppm of all analytes to check for instrumental drift.
(DOC)

## References

[1] Campbell KR (2003) Ecotoxicology of crocodilians. Appl Herpetol 1:45–163.

[2] Liu JH, Kueh CSW (2005) Biomonitoring of heavy metals and trace organics using the intertidal mussel *Perna viridis* in Hong Kong coastal waters. Mar Pollut Bull 51: 857–875. 15907944 10.1016/j.marpolbul.2005.04.014

[3] Kannan K, Agusa T, Perrotta E, Thomas NJ, Tanabe S (2006) Comparison of trace element concentrations in livers of diseased, emaciated and non-diseased southern sea otters from the California coast. Chemosphere 65:2160–2167. 16846630 10.1016/j.chemosphere.2006.06.003

[4] Rai PK (2008) Heavy metal pollution in aquatic ecosystems and its phytoremediation using wetland plants: an ecosustainable approach. Int J Phytoremediat 10: 133–160. 10.1080/15226510801913918 18709926

[5] Zhou Q, Zhang J, Fu J, Shi J, Jiang G (2008) Biomonitoring: an appealing tool for assessment of metal pollution in the aquatic ecosystem. Analy Chimica Acta 606: 135–150. 10.1016/j.aca.2007.11.018 18082645

[6] Rabalais NN, Turner RE, Dias RJ, Justic D (2009) Global change and eutrophication of coastal waters. ICES J Mar Sci 66: 1528–1537.

[7] Kelly EJ, Quaife CJ, Froelick GJ, Palmiter RD (1996) Metallothionein I and II protect against zinc deficiency and zinc toxicity in mice. J Nutr 126: 1782–1790. 8683339 10.1093/jn/126.7.1782

[8] Rink L, Gabriel P (2000) Zinc and the immune system. Proc Nutr Soc 59: 541–552. 11115789 10.1017/s0029665100000781

[9] Ibs K-H, Rink L (2003) Zinc-altered immune function. J Nutr 133: 1452–1456. 10.1093/jn/133.5.1452S 12730441

[10] Crossgrove J, Zheng W (2004) Manganese toxicity upon overexposure. NMR Biomed 17: 544–553. 15617053 10.1002/nbm.931 PMC3980863

[11] Sures B (2004) Environmental parasitology: relevancy of parasites in monitoring environmental pollution. TRENDS Parasitol 20(4): 170–177. 15099556 10.1016/j.pt.2004.01.014

[12] Nachev M, Zimmermann S, Rigaud T, Sures B (2010) Is metal accumulation in *Pomphorhynchus laevis* dependent on parasite sex or infrapopulation size? Parasitol 137: 1239–1248. 10.1017/S0031182010000065 20380766

[13] Orescanin V, Lovrencic I, Mikelic L, Barisic D, Matasin Z, Lulic S, Pezelj D (2006) Biomonitoring of heavy metals and arsenic on the east coast of the Middle Adriatic Sea using *Mytilus galloprovincialis* . Nuc Instrum Methods B 245: 495–500.

[14] Lafferty KD, Kuris Am (1999) How environmental stress affects the impacts of parasites. Limnol Oceano 44: 925–931.

[15] Halmetoja A, Valtonen ET, Koskenniemi E (2000) Perch (*Perca fluviatilis* L.) parasites reflect ecosystem conditions: a comparison of a natural lake and two acidic reservoirs in Finland. Int J Parasitol 30: 1437–1444. 11428333 10.1016/s0020-7519(00)00115-6

[16] Bergey L, Weis JS, Weis P (2002) Mercury uptake by the estuarine species *Palaemonetes pugio* and *Fundulus heteroclitus* compared with their parasites, *Probopyrus pandalicola* and *Eustrongylides* sp. Mar Pollut Bull 44: 1046–1050. 12474965 10.1016/s0025-326x(02)00154-6

[17] Schludermann C, Konecny R, Laimgruber S, Lewis JW, Schiemer F., et al (2003) Fish macroparasites as indicators of heavy metal pollution in river sites in Austria. Parasitol 126: S61–S69. 10.1017/s0031182003003743 14667173

[18] Sures B, Taraschewski M (1997) Intestinal fish parasites as heavy metal bioindicators: a comparison between *Acanthocephalus lucii* (Palaeacanthocephala) and the Zebra Mussel, *Dreissena polymorpha* . Bull Environ Con Toxicol 59: 14–21. 10.1007/s001289900437 9184035

[19] Sures B (2001) The use of fish parasites as bioindicators of heavy metals in aquatic ecosystems: a review. Aquatic Ecol 35: 245–255.

[20] Sures B, Taraschewski H, Haug C (1995) Determination of trace metals (Cd, Pb) in fish by electrothermal atomic absorption spectrometry after microwave digestion. Anal Chimica Acta 311: 135–139.

[21] Sures B, Jürges G, Taraschewski H (1998) Relative concentrations of heavy metals in the parasites *Safaris suum* (Nematoda) and *Fasciola hepatica* (Digenes) and their respective porcine and bovine definitive hosts. Int J Parasitol 28: 1173–1178. 9762561 10.1016/s0020-7519(98)00105-2

[22] Sures B, Slidell R, Taraschewski H (1999) Parasites as accumulation indicators of heavy metal pollution. Parasitol Today 15: 16–21. 10234173 10.1016/s0169-4758(98)01358-1

[23] Sures B, Zimmermann S, Sonntag C, Stüben D, Taraschewski H (2003) The acanthocephalan *Paratenuisentis ambiguous* as a sensitive indicator of the precious metals Pt and Rh emitted from automobile catalytic converters. Environ Pollut 122: 401–405. 12547529 10.1016/s0269-7491(02)00306-8

[24] Thielen F, Zimmerman S, Bask F, Taraschewski H, Sures B (2004) The intestinal parasite *Pomphorhynchus laevis* (Acanthocephalan) from barbell as a bioindicator for metal pollution in the Danube River near Budapest, Hungary. Environ Pollut 129: 421–429. 15016463 10.1016/j.envpol.2003.11.011

[25] Nachev M, Sures B (2009) The endohelminth fauna of barbell (*Barbus barbus*) correlates with water quality of the Danube River in Bulgaria. Parasitol 136: 545–552. 10.1017/S003118200900571X 19250599

[26] Lafferty KD, Dobson AP, Kuris AM (2006) Parasites dominate food web links. PNAS 103: 11211–11216. 16844774 10.1073/pnas.0604755103 PMC1544067

[27] Lafferty KD, Kuris AM. Parasitism and environmental disturbances In: Thomas F, Renaud F, Guegan J-F, editors. Parasitism and Ecosystems. Oxford: Oxford University Press; 2004.

[28] Burger J, Gochfeld M, Rooney AA, Orlando EF, Woodward AR, et al (2000) Metals and metalloids in tissues of American Alligators in three Florida lakes. Arch Environ Con Tox 38: 501–508. 10.1007/s002449910066 10787102

[29] Wu TH, Rainwater TR, Platt SG, McMurry ST, Anderson TA (2000) DDE in eggs of two crocodile species from Belize. J Agr Food Chem 48: 6416–6420. 11141295 10.1021/jf000321u

[30] Rainwater TR, Adair BM, Platt SG, Anderson TA, Cobb GP, et al (2002) Mercury in Morelet’s crocodile eggs from Northern Belize. Arch Environ Con Toxicol 42: 319–324. 10.1007/s00244-001-0020-7 11910460

[31] Rainwater T, Wu TH, Finger AG, Cañas JE, Yu L, et al (2007) Metals and organochlorine pesticides in caudal scutes of crocodiles from Belize and Costa Rica. Sci Tot Environ 373: 146–156. 10.1016/j.scitotenv.2006.11.010 17182086

[32] Pepper CB, Rainwater TR, Platt SG, Dever JA, McMurry ST, et al (2004) Organochlorine pesticides in chorioallantoic membranes of Morelet’s crocodile eggs from Belize. J Wildlife Dis 40: 493–500. 10.7589/0090-3558-40.3.493 15465717

[33] Lance VA, Horn TR, Elsey RM, de Peyster A (2006) Chronic incidental lead ingestion in a group of captive-reared and wild alligators (*Alligator mississippiensis*): possible contribution to reproductive failure. Comp Biochem Physiol C 142: 30–35. 10.1016/j.cbpc.2005.09.008 16290046

[34] Klassen CD (1978) Effect of metallothionein on hepatic disposition of metals. Am J Physiol-Gastr L 234: E47–E53. 10.1152/ajpendo.1978.234.1.E47 623248

[35] Otvos JD, Armitage IM (1980) Structure of the metal clusters in rabbit liver metallothionein. Proc Nat Sci 77(12): 7094–7096. 10.1073/pnas.77.12.7094 PMC350447 6938956

[36] Sato M, Kondoh M (2002) Recent studies on metallothionein: protection against toxicity of heavy metals and oxygen free radicals. Tohoku J Experiment Med 196: 9–22. 10.1620/tjem.196.9 12498322

[37] Ikemoto T, Kunito T, Tanaka H, Baba N, Miyazaki N, et al (2004) Detoxification mechanism of heavy metals in marine mammals and seabirds: interaction of selenium with mercury, silver, copper, zinc, and cadmium in liver. Arch Environ Con Toxicol 47: 402–413. 10.1007/s00244-004-3188-9 15386135

[38] Siva PRM, Hardaway CJ, Sneddon J (2010) Determination of cadmium, chromium, copper, iron, lead, and zinc in oysters from southwest Louisiana by inductively coupled plasma-optical emission spectrometry. Instrum Sci Technol 38: 448–457.

[39] Guillory G, Hardaway CJ, Merchant ME, Sneddon J (2011) Determination of selected metals in alligator (*Alligator mississippiensis*) tissues by inductively coupled plasma-optical emission spectrometry. Instrum Sci Technol 39: 368–373.

[40] R Development Core Team (2012) R: A language and environment for statistical computing R Foundation for Statistical Computing, Vienna, Austria ISBN 3-900051-07-0, URL http://www.R-project.org.

[41] Benjamini Y, Hochberg Y (1995) Controlling the false discovery rate: a practical and powerful approach to multiple testing. J Roy Stat Soc B 57: 289–300.

[42] Benjamini Y, Yekutieli D (2001) The control of the false discovery rate in multiple testing under dependency. Ann Stat 29: 1165–1188.

[43] Hopkins WA, Staub BP, Baionno JA, Jackson BP, Talent LJ (2005) Transfer of selenium from prey to predators in a simulated terrestrial food chain. Environ Pollut 134: 447–456. 15620590 10.1016/j.envpol.2004.09.010

[44] Law K, Halldorson T, Danell R, Stem G, Gewurtz S, et al (2009) Bioaccumulation and trophic transfer of some brominated flame retardants in a Lake Winnipeg (Canada) food web. Environ Toxicol 25: 2177–2186. 10.1897/05-500r.1 16916037

[45] Garcia-Hernandez J, Cadena-Cardenas L, Betancourt-Lozano M, Garcia-de-la-Parra LM, Garcia-Rico L, et al (2007) Total mercury content found in edible tissues of top predator fish from the Gulf of California, Mexico. Toxicol Environ Chem 89(3): 507–522.

[46] Chen CY, Serrell N, Evers DC, Fleishman BJ, Lambert KF, et al (2008) Methylmercury in marine ecosystems: from sources to seafood consumers. Environ Health Persp 116: 1706–1712. 10.1289/ehp.11211 PMC2599767 19079724

[47] Moss JC, Hardaway CJ, Richert JC, Sneddon J (2010) Determination of cadmium, copper, iron, nickel, lead, and zinc in crawfish (*Procambrus clarkii*) by inductively coupled plasma optical emission spectrometry: a study over the 2009 season in Southwest Louisiana. Microchem J 95: 5–10.

[48] Bell JU, Lopez JM (1985) Isolation and partial characterization of a cadmium-binding protein from the liver of alligators exposed to cadmium. Comp Biochem Phys C 82: 123–128. 10.1016/0742-8413(85)90218-x 2865051

[49] Flos R, Bas J, Hidalgo J (1986) Metallothionein in the liver of the small lizard *Podarcis muralis* . Comp Biochem Phys C 83: 93–98. 10.1016/0742-8413(86)90018-6 2869914

[50] Thomas P, Baer KN, White RB (1994) Isolation and partial characterization of metallothionein in the liver of the red-eared turtles (*Trachemys scripta*) following intraperitoneal administration of cadmium. Comp Biochem Phyisol C 107: 221–226.

[51] Bush AO, Fernandez JC, Esch GW, Seed JR. Parasitism: The diversity and ecology of animal parasites Cambridge: Cambridge University Press; 2001.

[52] Sandberg A-S, Andersson H, Hallgren B, Hasselblad K, Isaksson B, et al (1981) Experimental model for in vivo determination of dietary fibre and its effect on the absorption of nutrients in the small intestine. Brit J Nutri 45(2): 283–294. 10.1079/bjn19810105 6260129

[53] Pappenheimer JR, Reiss KZ (1987) Contribution of solvent drag through intercellular junctions to absorption of nutrients by the small intestine of the rat. J Membrane Biol 100(1): 123–136. 3430569 10.1007/BF02209145

[54] Diamond J (1991) Evolutionary design of intestinal nutrient absorption: enough but not too much. Physiol 6: 92–96.

[55] Frieden E (1973) The ferrous to ferric cycles in iron metabolism. Nutr Rev 31: 41–44. 4702209 10.1111/j.1753-4887.1973.tb05977.x

[56] LSUAg Center [Internet]. Summary of Agricultural and Natural Resources. [cited 2011 June and 2012 June]. Available from: http://www.lsuagcenter.com/agsummary/

[57] Louisiana Department of Revenue [Internet]. [cited 2012 October]. Available from: http://www.rev.state.la.us/sections/Publications/sr.aspx

[58] US Department of Commerce, Office of the U.S. Census Bureau. [Internet]. [2012 October] Available from: http://factfinder2.census.gov/faces/nav/jsf/pages/searchresults.xhtml?refresh=t#

[59] Pietrock M, Marcogliese DJ, Meinelt T, McLaughlin JD (2002) Effects of mercury and chromium upon longevity of *Diplostomum* sp. (Trematoda: Diplostomidae) cercariae. Parasitol. Res. 88: 225–229. 11954907 10.1007/s00436-001-0529-8

[60] Fairbrother A, Fix M, O’Hara T, Ribic CA (1994) Impairment of growth and immune function of avocet chicks form sites with elevated selenium, arsenic, and boron. J Wildlife Dis 30: 222–233. 10.7589/0090-3558-30.2.222 8028107

[61] Abernathy CO, Thomas DJ, Calderon RL (2003) Health effects and risk assessment of Arsenic. J Nutr 133: 15365S–1538S. 10.1093/jn/133.5.1536S 12730460

[62] Choong TSY, Chuah TG, Robiah Y, Gregory Koay FL, Azni I (2007) Arsenic toxicity, health hazards, and removal techniques from water: an overview. Desalination 217: 139–166.

[63] Coeurdassier M, Scheifler R, Mench M, Crini N, Vangronsveld J, et al (2010) Arsenic transfer and impacts on snails exposed to stabilized and untreated As- contaminated soils. Environ Pollut 158: 2078–2083. doi: 10.1016/j.envpol.2010.03.008 20362375 10.1016/j.envpol.2010.03.008

[64] Martin PA, McDaniel TV, Hughes KD, Hunter B (2011) Mercury and other heavy metals in free-ranging mink of the lower Great Lakes basin, Canada, 1998–2006. Ecotoxicol 20: 1701–1712 10.1007/s10646-011-0763-5 21874547

[65] Brožová A, Jankovská I, Miholová D, Scháňková Š, Truněčková J, Langrová I, Kudrnáčová M, Vadlejch J (2015) Heavy metal concentrations in the small intestine of red fox (*Vulpes vulpes*) with and without *Echinococcus multilocularis* infection. Environ Sci Pollut Res 22: 3175–3179. 10.1007/s11356-014-3733-7 25335764

[66] Altindag A, Yigit S (2005) Assessment of heavy metal concentrations in the food web of lake Beysehir, Turkey. Chemosphere 60: 552–556. 15950047 10.1016/j.chemosphere.2005.01.009

[67] Campbell LM, Norstrom RJ, Hobson KA, Muir DCG, Backus S, et al (2005) Mercury and other trace elements in a pelagic Arctic marine food web (Northwater Polynya, Baffin Bay). Sci Tot Environ 351–352: 247–263. 10.1016/j.scitotenv.2005.02.043 16061271

[68] Salla V, Hardaway CJ, Sneddon J (2011) Preliminary investigation of *Spartina alterniflora* for phytoextraction of selected heavy metals in soils from Southwest Louisiana. Microchem J 97: 207–212.

