# Supplementary material for: Biomonitoring Heavy Metal Pollution Using an Aquatic Apex Predator, the American Alligator, and Its Parasites
Source: PLoS One. 2015 Nov 10;10(11):e0142522. doi: 10.1371/journal.pone.0142522 (PMC4640838; doi:10.1371/journal.pone.0142522)
Supplement: S1 Table — (DOC) [file pone.0142522.s001.doc]

**S1 Table.** Geographic locations of collection sites in East Louisiana, West Louisiana, and Florida.

| **Region:** | **Collection Site:** | **Coordinates:** |
| --- | --- | --- |
| Louisiana East  Zone | Maurepas Swamp Wildlife Management Area WMA | 30°9'58"N, 90°38'40"E |
|  | Assumption Parish | 29°55'23"N, 91°5'53"E |
|  | Plaquemines Parish Government Delta | 29°14'35"N, 89°12'36"E |
|  | Abercrombie | 29°28'50"N, 89°41'38"E |
|  | Continental Land | 29°37'2”N, 90°53'22"E |
|  | St. Mary Parish | 29°43'10"N, 91°8'8"E |
|  | Assumption/St. Martin/Terrebonne Parishes | 29°42'3"N, 91°5'45"E |
|  | Morgan City | 29°42'20.77"N, 91°13'5.45"W |
|  | Raceland | 29°43'38.76"N, 90°35'56.31"W |
|  | Pierre Part | 29°57'47.55"N, 91°12'31.90"W |
|  | Patterson | 29°41'38.26"N, 91°18'1.42"W |
|  | Berwick | 29°41'40.75"N, 91°13'8.37"W |
|  | Pass-A-Loutre WMA | 29°10'31"N, 89°7'17"E |
| Louisiana West  Zone | Iberville Parish | 30°16'19"N, 91°26'34"E |
|  | Iberia Parish | 29°59'48"N, 91°42'46"E |
|  | Vermilion Corp | 29°58'29"N, 92°8'3"E |
|  | Pomme de Terre WMA | 31°3'2"N, 91°50'52"E |
|  | St. Mary LDWF | 30°0'1"N, 91°38'31"E |
|  | Belle Isle, LLC | 29°34'36"N, 89°34'27"E |
| Florida | Lake Sidney | 28°58'56"N, 81°31'43"E |
|  | Lake Loochloosa | 29°29'23"N, 82°8'33"E |
|  | Lake Apopka | 28°37'29"N, 81°37'31"E |
|  | Putnam | 29°37'35"N, 81°46'43"E |
|  | Crescent Lake | 29°28'57"N, 81°30'17"E |
|  | Lake Orange | 29°28'6"N, 82°11'16"E |
|  | Suwanee River | 29°28'12"N, 82°41'29"E |
|  | Lake George | 29°16'36"N, 81°35'1"E |
