# Supplementary material for: Biomonitoring Heavy Metal Pollution Using an Aquatic Apex Predator, the American Alligator, and Its Parasites
Source: PLoS One. 2015 Nov 10;10(11):e0142522. doi: 10.1371/journal.pone.0142522 (PMC4640838; doi:10.1371/journal.pone.0142522)
Supplement: S2 Table — (DOC) [file pone.0142522.s002.doc]

**S2 Table.** Summary of alligators from each region and site per year.

| Region: | Collection Site: | Year: |
| --- | --- | --- |
| Louisiana East  Zone | Maurepas Swamp Wildlife Management Area WMA | 2010: n = 0  2011: n = 3  2012: n = 0 |
|  | Assumption Parish | 2010: n = 0  2011: n = 1  2012: n = 0 |
|  | Plaquemines Parish Government Delta | 2010: n = 0  2011: n = 1  2012: n = 0 |
|  | Abercrombie | 2010: n = 0  2011: n = 2  2012: n = 0 |
|  | Continental Land | 2010: n = 0  2011: n = 4  2012: n = 0 |
|  | St. Mary Parish | 2010: n = 0  2011: n = 1  2012: n = 0 |
|  | Assumption/St. Martin/Terrebonne Parishes | 2010: n = 26  2011: n = 1  2012: n = 0 |
|  | Morgan City | 2010: n = 0  2011: n = 0  2012: n = 3 |
|  | Raceland | 2010: n = 0  2011: n = 0  2012: n = 1 |
|  | Pierre Part | 2010: n = 0  2011: n = 0  2012: n = 2 |
|  | Patterson | 2010: n = 0  2011: n = 0  2012: n = 3 |
|  | Berwick | 2010: n = 0  2011: n = 0  2012: n = 1 |
|  | Pass-A-Loutre WMA | 2010: n = 0  2011: n = 1  2012: n = 0 |
|  | Unidentified Location | 2010: n = 1  2011: n = 0  2012: n = 0 |
| Louisiana West  Zone | Iberville Parish | 2010: n = 0  2011: n = 8  2012: n = 0 |
|  | Iberia Parish | 2010: n = 0  2011: n = 15  2012: n = 0 |
|  | Vermilion Corp | 2010: n = 0  2011: n = 2  2012: n = 0 |
|  | Pomme de Terre WMA | 2010: n = 0  2011: n = 1  2012: n = 0 |
|  | St. Mary LDWF | 2010: n = 0  2011: n = 1  2012: n = 0 |
|  | Belle Isle, LLC | 2010: n = 0  2011: n = 3  2012: n = 0 |
|  | Unidentified Location | 2010: n = 1  2011: n = 0  2012: n = 0 |
| Florida | Lake Sidney | 2010: n = 0  2011: n = 1  2012: n = 0 |
|  | Lake Loochloosa | 2010: n = 0  2011: n = 4  2012: n = 1 |
|  | Lake Apopka | 2010: n = 0  2011: n = 2  2012: n = 3 |
|  | Putnam | 2010: n = 0  2011: n = 2  2012: n = 1 |
|  | Crescent Lake | 2010: n = 0  2011: n = 2  2012: n = 1 |
|  | Lake Orange | 2010: n = 0  2011: n = 3  2012: n = 0 |
|  | Suwanee River | 2010: n = 0  2011: n = 2  2012: n = 0 |
|  | Lake George | 2010: n = 0  2011: n = 2  2012: n = 2 |
|  | Unidentified Location | 2010: n = 0  2011: n = 1  2012: n = 0 |
