# Supplementary material for: Biomonitoring Heavy Metal Pollution Using an Aquatic Apex Predator, the American Alligator, and Its Parasites
Source: PLoS One. 2015 Nov 10;10(11):e0142522. doi: 10.1371/journal.pone.0142522 (PMC4640838; doi:10.1371/journal.pone.0142522)
Supplement: S3 Table — Samples 30 and 60 in each Tray are identified as Blanks as these were a standard solution containing 5 ppm of all analytes to check for instrumental drift. (DOC) [file pone.0142522.s003.doc]

**S3 Table.** Dry weight (g) of alligator liver samples, and parasite specimens from each year and geographic zone as organized in heavy metal analysis. Samples 30 and 60 in each Tray are identified as Blanks as these were a standard solution containing 5 ppm of all analytes to check for instrumental drift.

| **Tray 1** | **Year** | **Location** | **Sample** | **Dry Weight (g)** |
| --- | --- | --- | --- | --- |
| Sample 1 | 2011 | FL | Alligator | 3.2 |
| Sample 2 | 2011 | FL | Alligator | 3.5 |
| Sample 3 | 2011 | FL | Alligator | 2.1 |
| Sample 4 | 2011 | FL | Alligator | 6.7 |
| Sample 5 | 2011 | FL | Alligator | 0.0312 |
| Sample 6 | 2011 | FL | Alligator | 2.6 |
| Sample 7 | 2011 | FL | Alligator | 4.4 |
| Sample 8 | 2011 | FL | Alligator | 4 |
| Sample 9 | 2011 | FL | Alligator | 3.8 |
| Sample 10 | 2011 | FL | Alligator | 6.7 |
| Sample 11 | 2011 | FL | Alligator | 4.1 |
| Sample 12 | 2012 | FL | Intestinal nematodes | 0.1 |
| Sample 13 | 2012 | FL | Stomach Nematodes | 1.9 |
| Sample 14 | 2011 | FL | Alligator | 1.4 |
| Sample 15 | 2011 | FL | Alligator | 1.7 |
| Sample 16 | 2011 | FL | Alligator | 5.6 |
| Sample 17 | 2011 | FL | Alligator | 1.4 |
| Sample 18 | 2011 | FL | Alligator | 2.6 |
| Sample 19 | 2011 | FL | Alligator | 3.8 |
| Sample 20 | 2011 | FL | Alligator | 3.8 |
| Sample 21 | 2011 | FL | Alligator | 3.6 |
| Sample 22 | 2012 | LAE | Stomach Nematodes | 0.6 |
| Sample 23 | 2012 | LAE | Stomach Nematodes | 0.3 |
| Sample 24 | 2012 | FL | Intestinal nematodes | 0.02 |
| Sample 25 | 2012 | FL | Intestinal nematodes | 0.2 |
| Sample 26 | 2012 | FL | Stomach Nematodes | 0.02 |
| Sample 27 | 2012 | FL | Stomach Nematodes | 0.5 |
| Sample 28 | 2012 | FL | Intestinal nematodes | 0.1 |
| Sample 29 | 2012 | FL | Stomach Nematodes | 0.35 |
| Sample 30 | Blank | Blank | Blank | Blank |
| Sample 31 | 2012 | FL | Stomach Nematodes | 0.5 |
| Sample 32 | 2012 | LAE | Alligator | 3.9 |
| Sample 33 | 2012 | LAE | Alligator | 2.9 |
| Sample 34 | 2012 | LAE | Alligator | 4.1 |
| Sample 35 | 2012 | LAE | Alligator | 2.1 |
| Sample 36 | 2012 | LAE | Alligator | 2.4 |
| Sample 37 | 2012 | LAE | Alligator | 3.6 |
| Sample 38 | 2012 | LAE | Alligator | 1.9 |
| Sample 39 | 2012 | LAE | Alligator | 3.8 |
| Sample 40 | 2012 | LAE | Alligator | 1.9 |
| Sample 41 | 2012 | LAE | Alligator | 1.1 |
| Sample 42 | 2011 | LAE | Alligator | 6.5 |
| Sample 43 | 2011 | LAE | Alligator | 1.9 |
| Sample 44 | 2011 | LAE | Alligator | 5.2 |
| Sample 45 | 2011 | LAE | Alligator | 2.2 |
| Sample 46 | 2011 | LAE | Alligator | 5.8 |
| Sample 47 | 2011 | LAW | Alligator | 5.1 |
| Sample 48 | 2011 | LAE | Alligator | 2.9 |
| Sample 49 | 2010 | LAE | Alligator | 11.9 |
| Sample 50 | 2011 | LAE | Alligator | 2.7 |
| Sample 51 | 2010 | LAE | Alligator | 0.6 |
| Sample 52 | 2010 | LAE | Alligator | 5.4 |
| Sample 53 | 2010 | LAE | Alligator | 2.1 |
| Sample 54 | 2010 | LAE | Alligator | 2.8 |
| Sample 55 | 2012 | FL | Stomach Nematodes | 0.2 |
| Sample 56 | 2011 | LAE | Alligator | 1.2 |
| Sample 57 | 2011 | LAE | Alligator | 2.2 |
| Sample 58 | 2011 | LAW | Alligator | 4 |
| Sample 59 | 2011 | LAW | Alligator | 0.6 |
| Sample 60 | Blank | Blank | Blank | Blank |
| **Tray 2** |  |  |  |  |
| Sample 1 | 2011 | LAW | Alligator | 3 |
| Sample 2 | 2011 | LAW | Alligator | 2.5 |
| Sample 3 | 2011 | LAE | Alligator | 1.4 |
| Sample 4 | 2011 | LAE | Alligator | 2 |
| Sample 5 | 2011 | LAW | Alligator | 2.9 |
| Sample 6 | 2010 | LAE | Alligator | 4.9 |
| Sample 7 | 2011 | LAW | Alligator | 2 |
| Sample 8 | 2011 | LAW | Alligator | 3.6 |
| Sample 9 | 2011 | LAW | Alligator | 2.3 |
| Sample 10 | 2011 | LAW | Alligator | 2.1 |
| Sample 11 | 2011 | LAW | Alligator | 1.4 |
| Sample 12 | 2011 | LAW | Alligator | 2.1 |
| Sample 13 | 2011 | LAW | Alligator | 2.3 |
| Sample 14 | 2011 | LAW | Alligator | 4.3 |
| Sample 15 | 2011 | LAW | Alligator | 1.2 |
| Sample 16 | 2011 | LAW | Alligator | 1.7 |
| Sample 17 | 2011 | LAW | Alligator | 2.7 |
| Sample 18 | 2011 | LAW | Alligator | 3.3 |
| Sample 19 | 2011 | LAW | Alligator | 1.7 |
| Sample 20 | 2011 | LAW | Alligator | 1.4 |
| Sample 21 | 2011 | LAW | Alligator | 1.1 |
| Sample 22 | 2011 | LAW | Alligator | 2.1 |
| Sample 23 | 2011 | LAW | Alligator | 1.7 |
| Sample 24 | 2011 | LAE | Intestinal Trematodes | 0.008 |
| Sample 25 | 2011 | LAE | Intestinal Trematodes | 0.002 |
| Sample 26 | 2011 | LAW | Intestinal Trematodes | 0.002 |
| Sample 27 | 2011 | LAE | Intestinal Trematodes | 0.002 |
| Sample 28 | 2011 | LAW | Intestinal Trematodes | 0.001 |
| Sample 29 | 2010 | LAE | Alligator | 6.2 |
| Sample 30 | Blank | Blank | Blank | Blank |
| Sample 31 | 2010 | LAE | Alligator | 3.1 |
| Sample 32 | 2012 | FL | Alligator | 4.3 |
| Sample 33 | 2011 | LAW | Alligator | 7 |
| Sample 34 | 2010 | LAE | Alligator | 3.8 |
| Sample 35 | 2012 | FL | Stomach Nematodes | 0.5 |
| Sample 36 | 2010 | LAE | Alligator | 5.2 |
| Sample 37 | 2010 | LAE | Alligator | 2.4 |
| Sample 38 | 2012 | FL | Alligator | 6.3 |
| Sample 39 | 2011 | LAE | Alligator | 2.6 |
| Sample 40 | 2010 | LAE | Alligator | 3.3 |
| Sample 41 | 2010 | LAE | Alligator | 5.1 |
| Sample 42 | 2012 | FL | Alligator | 4.1 |
| Sample 43 | 2011 | LAW | Alligator | 3.4 |
| Sample 44 | 2011 | LAW | Alligator | 3.4 |
| Sample 45 | 2012 | FL | Alligator | 7 |
| Sample 46 | 2012 | FL | Alligator | 6.9 |
| Sample 47 | 2012 | FL | Alligator | 1 |
| Sample 48 | 2011 | LAW | Alligator | 3.7 |
| Sample 49 | 2011 | LAE | Alligator | 6.8 |
| Sample 50 | 2010 | LAE | Alligator | 3.3 |
| Sample 51 | 2010 | LAW | Alligator | 0.9 |
| Sample 52 | 2012 | FL | Stomach Nematodes | 0.4 |
| Sample 53 | 2010 | LAE | Stomach Nematodes | 3.1 |
| Sample 54 | 2010 | LAE | Stomach Nematodes | 1.3 |
| Sample 55 | 2010 | LAE | Stomach Nematodes | 1.6 |
| Sample 56 | 2012 | FL | Stomach Nematodes | 0.1 |
| Sample 57 | 2010 | LAE | Stomach Nematodes | 3.8 |
| Sample 58 | 2012 | FL | Stomach Nematodes | 0.2 |
| Sample 59 | 2010 | LAE | Alligator | 2.9 |
| Sample 60 | Blank | Blank | Blank | Blank |
| **Tray 3** |  |  |  |  |
| Sample 1 | 2010 | LAE | Alligator | 3.9 |
| Sample 2 | 2011 | LAW | Alligator | 2.2 |
| Sample 3 | 2010 | LAE | Alligator | 3.1 |
| Sample 4 | 2011 | LAW | Alligator | 3.7 |
| Sample 5 | 2012 | FL | Alligator | 4.1 |
| Sample 6 | 2012 | FL | Stomach Nematodes | 0.6 |
| Sample 7 | 2012 | FL | Alligator | 5.3 |
| Sample 8 | 2010 | LAE | Alligator | 4.3 |
| Sample 9 | 2011 | LAE | Alligator | 3.4 |
| Sample 10 | 2011 | LAE | Alligator | 3.1 |
| Sample 11 | 2012 | FL | Stomach Nematodes | 0.1 |
| Sample 12 | 2012 | FL | Stomach Nematodes | 0.1 |
| Sample 13 | 2012 | FL | Stomach Nematodes | 0.2 |
| Sample 14 | 2012 | FL | Stomach Nematodes | 0.2 |
| Sample 15 | 2010 | LAE | Alligator | 1 |
| Sample 16 | 2010 | LAE | Alligator | 2.5 |
| Sample 17 | 2010 | LAE | Alligator | 4.7 |
| Sample 18 | 2011 | LAW | Alligator | 1.4 |
| Sample 19 | 2010 | LAE | Alligator | 0.9 |
| Sample 20 | 2010 | LAE | Alligator | 1.7 |
| Sample 21 | 2011 | LAW | Lungs | 0.0705 |
| Sample 22 | 2011 | LAW | Lungs | 0.0156 |
| Sample 23 | 2011 | FL | Lungs | 0.0045 |
| Sample 24 | 2011 | FL | Lungs | 0.0065 |
| Sample 25 | 2011 | FL | Lungs | 0.0099 |
| Sample 26 | 2011 | FL | Lungs | 0.0216 |
| Sample 27 | 2011 | FL | Lungs | 0.0244 |
| Sample 28 | 2011 | LAW | Lungs | 0.0135 |
| Sample 29 | 2011 | FL | Lungs | 0.0167 |
| Sample 30 | Blank | Blank | Blank | Blank |
| Sample 31 | 2011 | LAE | Lungs | 0.0069 |
| Sample 32 | 2011 | FL | Lungs | 0.0126 |
| Sample 33 | 2011 | FL | Lungs | 0.0481 |
| Sample 34 | 2011 | FL | Stomach Nematodes | 2.292 |
| Sample 35 | 2010 | LAE | Intestinal Nematodes | 0.0121 |
| Sample 36 | 2010 | LAW | Stomach Nematodes | 0.0106 |
| Sample 37 | 2009 | LAW | Intestinal Nematodes | 0.0272 |
| Sample 38 | 2011 | LAE | Intestinal Nematodes | 0.0255 |
| Sample 39 | 2011 | LAW | Intestinal Nematodes | 0.0083 |
| Sample 40 | 2011 | LAW | Stomach Nematodes | 0.0599 |
| Sample 41 | 2011 | LAW | Lungs | 0.0034 |
| Sample 42 | 2011 | LAE | Stomach Nematodes | 0.3939 |
| Sample 43 | 2010 | LAW | Intestinal Nematodes | 0.004 |
| Sample 44 | 2011 | LAW | Lungs | 0.0013 |
| Sample 45 | 2011 | LAW | Lungs | 0.0149 |
| Sample 46 | 2011 | LAW | Lungs | 0.0056 |
| Sample 47 | 2011 | LAW | Lungs | 0.0058 |
| Sample 48 | 2011 | LAE | Intestinal Nematodes | 0.0239 |
| Sample 49 | 2011 | LAW | Intestinal Nematodes | 0.0246 |
| Sample 50 | 2011 | FL | Stomach Nematodes | 2.2526 |
| Sample 51 | 2011 | FL | Stomach Nematodes | 0.514 |
| Sample 52 | 2011 | FL | Stomach Nematodes | 0.1694 |
| Sample 53 | 2011 | FL | Stomach Nematodes | 2.4087 |
| Sample 54 | 2010 | LAW | Intestinal Nematodes | 0.0019 |
| Sample 55 | 2011 | FL | Lungs | 0.0568 |
| Sample 56 | 2011 | FL | Lungs | 0.0541 |
| Sample 57 | 2010 | LAW | Stomach Nematodes | 1.4213 |
| Sample 58 | 2010 | LAW | Stomach Nematodes | 0.1658 |
| Sample 59 | 2011 | LAE | Lungs | 0.0168 |
| Sample 60 | Blank | Blank | Blank | Blank |
| **Tray 4** |  |  |  |  |
| Sample 1 | 2011 | LAW | Lungs | 0.003 |
| Sample 2 | 2011 | LAE | Lungs | 0.0089 |
| Sample 3 | 2011 | LAW | Lungs | 0.0029 |
| Sample 4 | 2010 | LAE | Stomach Nematodes | 1.808 |
| Sample 5 | 2011 | LAW | Lungs | 0.0018 |
| Sample 6 | 2011 | LAE | Stomach Nematodes | 0.96 |
| Sample 7 | 2010 | LAE | Stomach Nematodes | 0.7045 |
| Sample 8 | 2010 | LAE | Stomach Nematodes | 0.0943 |
| Sample 9 | 2010 | LAW | Stomach Nematodes | 0.0111 |
| Sample 10 | 2011 | LAE | Lungs | 0.0037 |
| Sample 11 | 2010 | LAE | Stomach Nematodes | 0.0025 |
| Sample 12 | 2011 | LAW | Lungs | 0.007 |
| Sample 13 | 2010 | LAE | Stomach Nematodes | 0.019 |
| Sample 14 | 2011 | FL | Stomach Nematodes | 0.0519 |
| Sample 15 | 2011 | LAE | Intestinal Nematodes | 0.1044 |
| Sample 16 | 2009 | LAE | Intestinal Nematodes | 0.0397 |
| Sample 17 | 2010 | LAW | Stomach Nematodes | 0.0164 |
| Sample 18 | 2011 | FL | Lungs | 0.0424 |
| Sample 19 | 2011 | LAW | Lungs | 0.0085 |
| Sample 20 | 2011 | FL | Lungs | 0.0184 |
| Sample 21 | 2010 | LAE | Stomach Nematodes | 0.029 |
| Sample 22 | 2011 | FL | Stomach Nematodes | 1.4593 |
| Sample 23 | 2011 | FL | Lungs | 0.006 |
| Sample 24 | 2011 | FL | Lungs | 0.0106 |
| Sample 25 | 2011 | LAE | Lungs | 0.0086 |
| Sample 26 | 2011 | FL | Lungs | 0.0312 |
| Sample 27 | 2011 | LAW | Intestinal Nematodes | 0.0019 |
| Sample 28 | 2011 | LAW | Lungs | 0.0072 |
| Sample 29 | 2011 | LAE | Intestinal Nematodes | 0.0509 |
| Sample 30 | Blank | Blank | Blank | Blank |
| Sample 31 | 2009 | LAW | Intestinal Nematodes | 0.0084 |
| Sample 32 | 2010 | LAE | Stomach Nematodes | 0.0071 |
| Sample 33 | 2010 | LAW | Intestinal Nematodes | 0.003 |
| Sample 34 | 2011 | LAE | Stomach Nematodes | 0.0228 |
| Sample 35 | 2011 | LAW | Intestinal Nematodes | 0.007 |
| Sample 36 | 2011 | FL | Stomach Nematodes | 0.1229 |
| Sample 37 | 2011 | FL | Lungs | 0.0147 |
| Sample 38 | 2011 | FL | Stomach Nematodes | 0.0211 |
| Sample 39 | 2011 | LAW | Intestinal Nematodes | 0.0013 |
| Sample 40 | 2010 | LAE | Intestinal Nematodes | 0.0433 |
| Sample 41 | 2011 | LAE | Intestinal Nematodes | 0.0038 |
| Sample 42 | 2010 | LAW | Stomach Nematodes | 0.0019 |
| Sample 43 | 2011 | FL | Stomach Nematodes | 0.1099 |
| Sample 44 | 2011 | LAW | Stomach Nematodes | 0.0986 |
| Sample 45 | 2011 | FL | Stomach Nematodes | 0.1173 |
